# Nutrition in the prevention and treatment of pelvic inflammatory disease: a review

**DOI:** 10.3389/fnut.2026.1802637

**Published:** 2026-04-01

**Authors:** Guanglong Wang, Suo Zhang

**Affiliations:** College of Traditional Chinese Medicine, Inner Mongolia Medical University, Hohhot, China

**Keywords:** dietary antioxidants, dietary choline intake, dietary fiber intake, dietary minerals, nutrition, pelvic inflammatory disease

## Abstract

Pelvic inflammatory disease (PID) is a common gynecological condition that causes inflammation in the upper reproductive tract. It affects millions of women globally each year and can lead to long-term complications such as infertility, ectopic pregnancy, and chronic pelvic pain. Although conventional PID treatments rely mainly on antibiotics, nutrition is crucial for preventing and treating PID because it can modulate immune responses, reduce inflammation, and promote tissue repair. This review aims to summarize the link between nutrition and PID by analyzing how dietary antioxidants such as vitamins C and E and polyphenols protect against oxidative stress and inhibit proinflammatory mediators in pelvic tissues. This study also explores the roles of trace minerals including magnesium, phosphorus, and copper in supporting immune cell function and maintaining mucosal barrier integrity. Other nutrients, including choline and dietary fiber, are also discussed. By combining clinical and preclinical research, this review emphasizes the potential of targeted nutrition as an adjunct to conventional PID management. Finally, it highlights research gaps, such as the lack of large-scale randomized controlled trials for optimal nutrient dosages, and suggests future directions for developing evidence-based nutritional guidelines for women at risk of or affected by PID.

## 1 Introduction

Pelvic inflammatory disease (PID) is a collective term for a group of conditions caused by infections of the female upper reproductive tract, primarily including endometritis, salpingitis, tubo-ovarian abscess, and pelvic peritonitis (1–3). This common gynecological infectious disease is caused mainly by ascending pathogens such as *Neisseria gonorrhoeae* and *Chlamydia trachomatis* (4–6). If PID is not treated promptly and thoroughly, it can lead not only to sequelae such as chronic pelvic pain, infertility, and ectopic pregnancy (1–3) but also to pelvic malignancies (ovarian cancer, cervical cancer, vaginal cancer, etc.) (7–10), sepsis (11), bacteremia (12), intestinal obstruction (13–15), and depression/anxiety (16). Surveys indicate that PID affects 4%–12% of women of reproductive age globally (17), with a higher prevalence among sexually active young women (18, 19). In the United States alone, approximately 500,000 to 1 million new cases are diagnosed annually, with the average treatment cost per case reaching $3,025 (20, 21). However, owing to atypical symptoms in some patients or delayed medical consultation, the actual incidence of PID may be underestimated (22, 23). Currently, PID treatment relies primarily on antibiotics, but their overuse may lead to the development of drug resistance and the inability to completely eliminate inflammation or prevent sequelae (24). Therefore, identifying a safe and effective adjunctive treatment method is crucial.

In recent years, dietary intervention has gradually attracted researchers’ attention as a non-pharmacological therapy. Its role in the adjunctive treatment of various diseases has become increasingly significant, with evidence demonstrating its potential therapeutic effects on multiple conditions (25). Several studies have indicated that dietary interventions play a crucial role in women’s reproductive health. For example, dietary supplements can alleviate pain associated with endometriosis (26, 27), a ketogenic diet may regulate ovarian function in women with polycystic ovary syndrome (28), dietary fiber intake is negatively correlated with infertility risk (29), and vitamin B6 may reduce the risk of endometriosis (30). Notably, existing research has identified associations between specific dietary patterns or nutrient intake adjustments and the incidence risk and severity of PID. One survey revealed a close correlation between dietary interventions and PID prevalence. After adjusting for confounding factors, dietary interventions showed an inverse linear association with PID risk (i.e., greater intervention adherence correlated with lower risk), suggesting therapeutic potential for PID (31). However, despite the large number of relevant studies, no systematic review on dietary factors and PID has been published. Therefore, this study aims to fill this gap by systematically elucidating the role of dietary nutrients in the adjunctive treatment of PID and their potential mechanisms of action to provide scientific evidence for clinical practice (Table 1).

**TABLE 1:** Dietary factors under investigation and their role in pelvic inflammatory disease (PID).

Nutrition category	Key nutrients	Biological role in PID
Dietary antioxidants	Vitamin A	Anti-inflammatory; antioxidant (40, 41)
	Vitamin C	Anti-inflammatory; antioxidant; cell protection (42, 43)
	Vitamin E	Anti-inflammatory; antioxidant; cell protection (44)
	Carotenoids	Antioxidant; regulates microbial balance (45)
	Selenium	Anti-inflammatory; antioxidant; regulates immune cells (46)
	Zinc	Anti-inflammatory; antioxidant; regulates immune cells (47)
Dietary minerals	Magnesium	Anti-inflammatory; antioxidant; regulates immune cells (55–57)
	Phosphorus	Energy metabolism; anti-inflammatory; antioxidant; cell protection (59–62)
	Copper	Anti-inflammatory; antioxidant; regulates immune cells; regulates estrogen levels (66–71)
Other dietary nutrients	Choline	Anti-inflammatory; regulates microbial balance; alleviates inflammatory hyperalgesia (75–83)
	Fiber	Anti-inflammatory; regulates microbial balance; regulates immune cells (87–90)

## 2 Nutrition and PID

Current research on the association between nutritional status and the risk of PID onset remains limited and insufficiently comprehensive. However, multiple observational studies examining both overall dietary patterns and individual nutrients have provided findings that support the potential role of nutritional interventions in this field (32).

### 2.1 Dietary antioxidants

A diet rich in antioxidants (including vitamins A, C, E, carotenoids, selenium, and zinc) has been shown to aid in managing chronic pelvic pain, including pain associated with PID (32). A 2025 nationwide survey and Mendelian randomization study revealed a significant negative correlation between standardized dietary antioxidant capacity index (cDaI) levels in American women and the incidence risk of PID (33). Specifically, each one-unit increase in the cDaI was associated with a 5% reduction in the probability of developing PID (33). This effect was most pronounced among women aged 40–49 years, those with a BMI of 25–30 kg/m^2^, and women without hypertension (33). In a multivariate logistic regression model adjusted for individual antioxidant components, zinc intake was found to be significantly negatively associated with PID risk (33). The data indicated that for each unit increase in zinc intake, the risk of PID decreased by 4% (33).

The cDaI is a well-established and reliable nutritional assessment tool for evaluating the overall antioxidant status of an individual’s diet (34). This index provides a composite score encompassing multiple dietary antioxidants, including vitamins A, C, E, carotenoids, selenium, and zinc (34). The cDaI has been applied in dietary studies focused on various gynecological conditions (35–39). The results from a 2025 cross-sectional study revealed a significant negative linear correlation between the cDaI and endometriosis among U.S. adult women, suggesting that increased dietary intake of antioxidant-rich foods may help prevent endometriosis (37). Shao et al. reported that increased dietary antioxidant intake may be associated with a reduced risk of female infertility (35). A study examining the association between antioxidant-rich diets and the risk of hyperemesis gravidarum in pregnant Chinese women revealed the key role of antioxidant-rich foods in preventing hyperemesis gravidarum (39).

Given the pivotal role of antioxidants in anti-inflammatory mechanisms, dietary modulation rich in antioxidants may hold clinical significance for PID. Studies indicate that vitamin A exerts anti-inflammatory effects by reversing inflammation-related abnormalities induced by its deficiency. This occurs through the inhibition of NF-κB activation, the reduction of oxidative stress, the regulation of antioxidant enzyme activity, and the suppression of iNOS and COX-2 expression (40, 41). Vitamin C exerts anti-inflammatory effects by inhibiting the NF-κB signaling pathway (which reduces ROS production, suppresses IKK phosphorylation, and decreases NF-κB DNA-binding activity), modulating cytokine balance (which reduces IL-6 and TNF-α and upregulates anti-inflammatory factors such as IL-10), inhibiting PLA2 activity to reduce inflammatory mediator release, and maintaining cell membrane integrity while suppressing apoptosis by reducing oxidative stress (42, 43). The anti-inflammatory mechanisms of vitamin E involve both direct and indirect actions. Direct effects prevent lipid peroxidation and associated cell membrane damage, thereby maintaining membrane integrity and signaling pathways to directly influence immune cell function. Indirect effects regulate inflammatory mediator production—such as the suppression of proinflammatory cytokines and PGE2—to reduce inflammatory responses (44). Carotenoids can upregulate the relative abundance of beneficial bacterial genera such as *Akkermansia*, *Candidatus Stoquefichus*, and *Faecalibaculum* while downregulating harmful genera such as *Alloprevotella* and *Helicobacter*. This suppresses inflammatory cytokines and reduces inflammatory responses, thereby promoting uterine recovery (45). Selenium primarily exerts anti-inflammatory effects through selenoproteins. These proteins modulate inflammation via multiple pathways, including regulating redox signaling, influencing calcium signaling pathways, controlling cytokine production, promoting immune cell differentiation, protecting endoplasmic reticulum function, and modulating signaling molecule activity (46). Furthermore, the anti-inflammatory mechanisms of zinc primarily include the suppression of iNOS enzyme expression, the inhibition of proinflammatory cytokine release, the suppression of MPO activity, the inhibition of the NF-κB pathway, and the suppression of mast cell degranulation (47, 48). Therefore, the multicomponent nature and diverse anti-inflammatory mechanisms of antioxidants confer unique advantages and therapeutic potential for dietary antioxidants in the adjunctive treatment of PID.

### 2.2 Dietary minerals

#### 2.2.1 Magnesium

Magnesium is an essential macromineral (not a trace element) in the human body. An adult contains approximately 25 g of magnesium, with 60% stored in bones, 39% distributed in muscles and soft tissues, and only 1% present in the blood. It is involved in more than 300 enzymatic reactions within the body and is the fourth most important mineral in humans and the second most abundant intracellular cation after potassium (49–51). A cross-sectional study from 2015 to 2018 revealed a significant association between increased dietary magnesium intake and a reduced incidence of PID (52). This association was particularly pronounced in specific populations: older women, members of low- to middle-income households, individuals who were normal weight or overweight, non-smokers, those without diabetes, and those with irregular menstrual cycles, all of whom reported a significant negative correlation between dietary magnesium intake and PID risk (52). Furthermore, magnesium supplementation has been demonstrated to effectively alleviate pelvic pain symptoms (53, 54). Therefore, for women at high risk for PID, achieving the National Institutes of Health (NIH)-recommended daily intake of 300 mg of magnesium is advised (52).

Although the potential benefits of magnesium for PID have been supported by some studies, its specific mechanism of action remains poorly understood (52). Magnesium exerts complex and diverse effects within the body and potentially achieves anti-inflammatory effects through multiple pathways, including immune cell regulation, intervention in inflammatory signaling pathways, modulation of oxidative stress and endothelial function, and direct regulation of inflammatory markers (55–57). First, magnesium modulates macrophage polarization, promoting their shift toward the anti-inflammatory M2 phenotype. This reduces the expression of proinflammatory M1 markers while increasing the expression of anti-inflammatory M2 markers (57). Second, magnesium directly decreases serum concentrations of core inflammatory markers such as CRP and increases the levels of nitric oxide (NO), which has anti-inflammatory and cardiovascular protective effects (56). Third, magnesium reduces the release of proinflammatory cytokines such as TNF-α and IL-1 by inhibiting inflammatory signaling pathways such as the NF-κB pathway (55, 56). Finally, magnesium improves oxidative stress states, reduces inflammation-related oxidative damage, and directly modulates other inflammation-related markers, including fibrinogen and acid phosphatase 5b (56).

#### 2.2.2 Phosphorus

Phosphorus is an essential macromineral in the human body, with approximately 85% present in the form of phosphates within bones and teeth. The remainder is distributed in intracellular and extracellular fluids and participates in various physiological processes (58). An analysis of data from the 2015–2018 U.S. National Health and Nutrition Examination Survey revealed a significant negative correlation between dietary phosphorus intake and the prevalence of PID (59). That is, higher dietary phosphorus intake was associated with a lower prevalence of PID (59). Studies indicate that each 1 mg/day increase in phosphorus intake reduces the risk of PID by 0.1%, with the protective effect of dietary phosphorus being more pronounced in the elderly population (59).

The mechanism underlying the effects of phosphorus on PID remains unclear, and even the mechanisms through which phosphorous affects inflammatory diseases in general are understudied. Mai et al. hypothesized that phosphorus may regulate PID through multiple biological pathways, primarily involving energy metabolism optimization, immune modulation, and oxidative stress alleviation (59–62). Furthermore, phosphorus primarily participates in physiological functions within the body as inorganic phosphate. Because phosphate is a crucial component of phospholipids in cell membranes, dietary phosphorus may promote the repair of damaged cell membranes, maintain cellular integrity, and reduce the leakage of inflammatory mediators.

#### 2.2.3 Copper

Copper is an essential trace element in the human body. Although trace elements constitute only a tiny proportion of the diet, they play a crucial role in maintaining normal bodily functions and health (48, 63). Both excessive and insufficient intakes of trace elements can disrupt the body’s inherent metabolic balance. A 2023 study confirmed a significant negative correlation between dietary copper intake and PID, indicating that lower dietary copper intake is associated with an increased risk of PID (64). This research provides important reference values for establishing recommended dietary copper intake for women with PID. Women should ensure that their diets contain sufficient copper to meet the recommended daily intake (0.9 mg/day) to reduce the risk of PID (64). Adequate copper intake may be particularly important for elderly and overweight women with PID (64). A 2015 review highlighted poultry as a good source of essential minerals such as copper, with 100 g of chicken thigh providing 1.4 mg of copper—which is comparable to the amount found in adult male beef (1.3 mg) (65). Moreover, copper in poultry is highly bioavailable, similar to other nutrients, and enhances the absorption of non-heme iron from other foods when it is consumed alongside meat (65).

According to current research on the anti-inflammatory mechanisms of copper, its potential mechanisms against PID may include the following: the inhibition of key inflammatory enzymes such as COX-1/2 and LOX to reduce the production of inflammatory mediators (66); the modulation of oxidative stress, such as by reducing ROS-induced cellular stress pathways to lower inflammatory responses (67); and interaction with inflammatory signaling pathways such as NF-κB to suppress the expression of proinflammatory cytokines such as TNF-α (68). Studies have indicated that copper can also induce anti-inflammatory cytokines (69), inhibit apoptosis (66), influence the activation and differentiation of immune cells (e.g., macrophages and T cells) (70), and suppress excessive immune responses, thereby alleviating inflammation. Furthermore, dietary copper intake has been demonstrated to be correlated with inflammatory diseases in women and serum estradiol levels (71), providing theoretical support for the potential indirect role of copper in preventing and treating PID through the regulation of hormone levels.

### 2.3 Other dietary nutrients

#### 2.3.1 Choline

Choline, an essential nutrient with a recommended daily intake of 0.425 g/day for women, not only serves as a crucial component of cell membranes but also participates in processes such as fat metabolism, nerve transmission, and methylation (72). In recent years, research on the relationship between choline and PID has gradually emerged. A cross-sectional study conducted between 2013 and 2018 confirmed that higher choline intake was negatively associated with PID risk (73). This finding suggests that adequate choline intake may help prevent PID among high-risk women. Subgroup analyses within this project revealed that among hypertensive participants, higher choline intake was linked to a lower incidence of PID (73). Blood pressure status emerged as a significant effect modifier, revealing distinct patterns between hypertensive and non-hypertensive subgroups. A threshold (bifurcated) relationship was observed in non-hypertensive individuals, with an overall inflection point at 0.41 g/day, whereas the threshold was lower (0.29 g/day) for normotensive subjects (73). Research has indicated that choline-rich diets primarily include foods such as eggs, red meat, milk, and cheese (74).

As a selective agonist of α7 nicotinic acetylcholine receptors (α7nAChRs), choline primarily exerts anti-inflammatory and analgesic effects through peripheral pathways (75), effectively alleviating PID and pelvic pain. On the one hand, choline activates α7nAChRs, thereby inhibiting the release of proinflammatory cytokines such as TNF-α and IL-6. It also hyperpolarizes primary nociceptive neurons by activating the NO/cGMP/ATP-sensitive potassium channel pathway, reducing inflammatory hyperalgesia (75). This process does not affect neutrophil migration or cytokine/chemokine production. On the other hand, acetylcholine—a metabolite of choline—can reduce proinflammatory cytokine release through cholinergic anti-inflammatory pathways, including the vagus nerve-immunoregulatory axis (76–78). Another metabolite, betaine, enhances tight junction protein expression in the gut and inhibits inflammatory pathways (79, 80). Concurrently, it increases the abundance of beneficial gut microbiota, protecting intestinal mucosal barrier function (81, 82) and indirectly reducing inflammation risk. Furthermore, choline may promote the release of IL-10 by activating the nAChR/ERK pathway (83), thereby further suppressing inflammatory responses.

#### 2.3.2 Fiber

Dietary fiber, as an essential nutrient for the human body, cannot be broken down and absorbed by digestive enzymes. However, it plays an irreplaceable role in maintaining intestinal health and regulating immune function. Current dietary guidelines recommend that adult women consume 25–32 g/day of fiber (84). In a study examining the association between dietary fiber intake and PID in women, an L-shaped negative correlation was observed between fiber intake and PID prevalence (85). In a fully adjusted model, each additional gram per day of dietary fiber intake was associated with a 5% reduction in PID risk (85). Subgroup analyses indicated that this protective effect was particularly pronounced among women aged 33 years or older, non-Hispanic whites, those with higher education levels, overweight individuals, non-smokers, women without diabetes, and postmenopausal women (85). Sensitivity analyses further validated the robustness of these findings, suggesting that increasing dietary fiber intake—particularly to levels exceeding 19.45 g/day—may help reduce the risk of PID among women (85). Notably, fiber-rich foods (such as whole grains, legumes, nuts, and leafy greens) are also important sources of magnesium (86).

Research has indicated that dietary fiber modulates immune function and reduces inflammation by regulating the gut microbiota (87). Soluble dietary fiber, in particular (88), can be fermented by probiotics (*Bifidobacterium*, *Lactobacillus*, etc.) to produce short-chain fatty acids (SCFAs, such as butyrate and propionate). This decreases the intestinal pH to inhibit pathogens while increasing the expression of tight junction proteins (occludin and claudins) to strengthen the intestinal barrier and block endotoxin entry into the bloodstream (88–90). Second, SCFAs inhibit the proinflammatory NF-κB pathway by activating GPR41/43 receptors or promoting the expression of the Foxp3 gene by acting as HDAC inhibitors, driving regulatory T-cell differentiation and IL-10 secretion. This suppresses Th1/Th17 responses and the release of proinflammatory factors such as TNF-α and IL-6. Concurrently, SCFAs synergistically regulate the bile acid-FXR axis and AhR signaling pathways, resulting in the formation of a systemic anti-inflammatory network (88).

## 3 Discussion

Dietary nutrients (including antioxidant diets, magnesium, phosphorus, copper, choline, dietary fiber, and other components) influence the onset and progression of PID through multiple mechanisms. These include regulating immune-inflammatory pathways, maintaining gut microbiota homeostasis, and alleviating oxidative stress. Consequently, they offer potential dietary intervention strategies for PID prevention and treatment. However, several critical limitations in current research must be explicitly acknowledged: first, most observed associations between dietary nutrients and PID are derived from cross-sectional or observational studies, which cannot establish causality; second, evidence from randomized controlled trials remains limited, particularly regarding long-term effects and optimal nutrient dosages; third, the precise causal relationships and optimal dosing regimens for specific nutrients in PID management have not yet been firmly established. Additionally, existing research remains insufficient in elucidating the underlying mechanisms of dietary nutrients, population studies face limitations, and the differential mechanisms among distinct subgroups remain unclear. Therefore, future research should prioritize high-quality randomized controlled trials to validate the practical efficacy of dietary interventions and determine optimal nutrient dosages. Concurrently, multiomics technologies should be employed for in-depth analysis of the complex interaction network among nutrients, the gut microbiota, and the immune system. Additionally, personalized dietary guidance based on individual metabolic characteristics should be actively explored to advance the individualized and scientific development of PID prevention and treatment strategies.
